# Co-Spray-Dried Macitentan–Tadalafil with Leucine Microparticles for Inhalable Delivery in Pulmonary Arterial Hypertension

**DOI:** 10.3390/pharmaceutics18020155

**Published:** 2026-01-25

**Authors:** Chang-Soo Han, Jin-Hyuk Jeong, Hyeon Woo Moon, Yechan Song, Chun-Woong Park

**Affiliations:** 1 Department of Pharmacy, Chungbuk National University, Cheongju 28644, Republic of Korea; hsoo0805@naver.com (C.-S.H.); jinddong92@gmail.com (J.-H.J.); mhw2@dkpharm.co.kr (H.W.M.); yechan0205@naver.com (Y.S.); 2 Dongkook Pharmaceutical Co., Ltd., Gwacheon 13840, Republic of Korea

**Keywords:** macitentan, tadalafil, spray drying, dry powder inhalation, combination therapy, pulmonary arterial hypertension

## Abstract

**Background/Objectives**: This study developed a macitentan (MAC)–tadalafil (TAD) dry powder inhalation preparation using suspension-based spray drying to enhance pulmonary delivery and reduce systemic exposure to oral combination therapy in patients with pulmonary arterial hypertension (PAH). **Methods**: MAC–TAD composite powders were prepared by physically mixing or spray-drying aqueous ethanol suspensions at various MAC:TAD ratios. The lead M2-T8 was co-spray-dried with 5, 25, or 50% (*w*/*w*) L-leucine. **Results**: Spray-dried formulations exhibited narrower and more uniform particle size distributions (Dv50 2–6 µm; Dv90~10 µm) and higher emitted dose values than the physical mixtures. In the M2-T8 spray-dried formulation, TAD exhibited an elevated fine particle dose (FPD) (3073.45 ± 1312.30 μg), demonstrating improved aerosolization relative to the physical mixture, even outperforming the TAD-higher M1-T9 formulation (2896.83 ± 531.38 μg), suggesting that favorable interparticle adhesive interactions were developed during co-drying. The incorporation of 25% L-leucine produced the greatest improvement in dispersibility, increasing the FPD by ~31% for MAC and 17% for TAD, whereas excessive L-leucine (50%) reduced the aerosol performance. Powder X-ray diffraction and differential scanning calorimetry confirmed the retention of the MAC and TAD crystallinities, with L-leucine remaining either amorphous or partially crystalline. **Conclusions**: Suspension-based spray drying yielded MAC–TAD composite formulations with improved uniformity and aerosol performance. The optimized 2:8 formulation containing 25% L-leucine demonstrated the most efficient pulmonary deposition, supporting its potential as an inhaled combination therapy for the treatment of PAH.

## 1. Introduction

Pulmonary arterial hypertension (PAH) is a progressive and life-threatening disorder characterized by abnormally elevated pressure in the pulmonary arteries [1]. Patients commonly experience dyspnea, chest pain, syncope, fatigue, and peripheral edema, with a higher prevalence reported in females [2,3]. Although PAH is classified as a rare disease, epidemiological analyses have estimated a global prevalence of approximately 15–60 cases per million people and an age-standardized prevalence rate of 2.3 per 100,000 individuals [4,5]. Recent data indicate that PAH will account for over 22,000 deaths worldwide in 2021, underscoring its substantial clinical burden despite advances in therapy [6]. Currently, PAH management involves endothelin receptor antagonists (ERAs), phosphodiesterase-5 (PDE-5) inhibitors, soluble guanylate cyclase stimulators, and prostacyclin-pathway agents [7]. Despite the availability of multiple pharmacological options, PAH remains incurable, and disease progression is frequently associated with insufficient pulmonary drug exposure and dose-limiting systemic adverse effects. Current guidelines recommend early combination therapy rather than monotherapy to improve long-term clinical outcomes [8,9,10].

Macitentan (MAC), marketed as Opsumit^®^, is an orally administered dual ERA targeting endothelin receptors, ETA and ETB, thereby counteracting endothelin-1-mediated vasoconstriction and vascular remodeling [11]. Although MAC demonstrates sustained receptor occupancy and proven clinical benefits in delaying PAH progression, its oral administration requires systemic exposure and is associated with adverse effects such as hepatotoxicity and anemia [12]. Furthermore, oral dosing necessitates relatively high systemic drug levels to achieve sufficient pulmonary exposure, raising safety concerns during long-term therapy.

Tadalafil (TAD), commercially available as Adcirca^®^ and Cialis^®^, is a PDE-5 inhibitor that prevents the degradation of cyclic guanosine monophosphate (cGMP) within the pulmonary vascular smooth muscle, resulting in vasodilation and reduced pulmonary vascular resistance [13]. The long half-life of TAD provides pharmacokinetic advantages; however, oral dosing produces delayed-onset and systemic side effects [14,15]. These limitations highlight the potential benefits of localized pulmonary delivery, which may enable lower nominal doses while achieving therapeutically relevant concentrations at the site of action.

Combination therapy with an ERA and a PDE-5 inhibitor is recommended by the World Health Organization functional class guidelines and has been shown to reduce disease progression, mortality, and hospitalization compared with monotherapy [8,16]. A fixed-dose combination of MAC (10 mg) and TAD (40 mg) has recently demonstrated superior clinical outcomes compared with either agent alone; however, increased systemic adverse effects have been reported [17]. Converting this combination to an inhaled formulation may preserve the therapeutic advantages of combination therapy, while reducing systemic exposure and achieving faster pulmonary onset.

Dry powder inhalation (DPI) preparations deliver drugs directly to the lungs, enabling rapid pharmacological action, enhanced pulmonary selectivity, and reduced systemic absorption [18,19,20]. Among manufacturing methods, the spray drying process is particularly advantageous because it allows co-encapsulation of multiple active ingredients within a single composite particle, facilitating synchronized deposition and potential synergistic action at the same pulmonary site [21,22,23]. Leucine, which is widely used as a force-control agent, improves aerosol performance by forming a surface-modifying, dispersibility-enhancing coating on spray-dried particles [24]. By reducing interparticle cohesion and enhancing aerosolization efficiency, L-leucine modification may further increase the fraction of the emitted dose reaching the deep lung, thereby improving therapeutic efficiency and safety margins.

In this study, MAC–TAD composite particles were prepared via spray drying of drug suspensions to generate ratio-dependent composite agglomerates. The physicochemical characteristics and aerodynamic performance of the formulations were evaluated at different MAC:TAD ratios. Furthermore, L-leucine was incorporated into the selected formulation to further improve inhalation efficiency, and the resulting DPI systems were systematically assessed. The delivered dose per inhalation and its potential clinical relevance relative to approved oral doses were considered to place the findings within a translational framework.

To the best of our knowledge, there have been few studies on the preparation of dual-drug DPI systems combining an endothelin receptor antagonist (MAC) and a PDE-5 inhibitor (TAD) using a co-spray-drying strategy. This work provides translational insight into how inhaled combination therapy may achieve clinically meaningful pulmonary exposure while potentially reducing systemic toxicity.

## 2. Materials and Methods

### 2.1. Materials

MAC was provided by Hetero Infrastructures SEZ, Ltd. (Anakapalli, India). TAD was obtained from MSN Organics Pvt., Ltd. (Bibinagar, India). L-Leucine and calcium chloride were purchased from Samchun Pure Chemicals Co., Ltd. (Pyeongtaek, Republic of Korea). Three hydroxypropyl methylcellulose capsules were obtained from Suheung (Cheongju, Republic of Korea). Methanol and acetonitrile (High-performance liquid chromatography grade) were purchased from Honeywell Burdick & Jackson, Ltd. (Muskegon, MI, USA). All other chemicals were of analytical grade and were used as received. All experiments were performed using Milli-Q distilled water (Merck Millipore, Burlington, MA, USA).

### 2.2. Physical Mixture of MAC and TAD

As shown in Table 1, MAC and TAD were accurately weighed and transferred into 2 mL microtubes (Corning, Union City, CA, USA). The mixtures were vortexed using a Vortex Genie 2 (Scientific Industries, Bohemia, NY, USA) at 3200 rpm for 2 min to obtain the formulations.

### 2.3. Spray Drying of MAC and TAD

The compositions and spray-drying conditions are listed in Table 2. MAC and TAD were accurately weighed and dissolved in 20% (*v*/*v*) aqueous ethanol. Ethanol was selected as a relatively safe solvent and preliminary screening was conducted using ethanol concentrations of 20%, 50%, and 80%. Although higher concentrations (50% and 80%) were initially tested, the final particles exhibited aggregation, resulting in relatively large Dv90 values of 35.87 ± 2.63 µm and 15.37 ± 1.76 µm, respectively, compared with 9.9 ± 0.1 µm at 20%. Based on these results, 20% ethanol was selected for further experiments. The mixture was then stirred for 1 h to obtain a homogeneous suspension. The resulting suspension was then subjected to spray drying. Spray drying was performed using an inlet temperature of 120 °C, a spray air flow of 35 m^3^/h, an aspirator rate of 100%, a pump rate of 10 mL/min, and a nozzle clean interval of 2 min to obtain the powder.

### 2.4. Scanning Electron Microscopy and Particle Size Distribution

Morphology and size distributions of the prepared formulations were characterized. The morphology was examined using scanning electron microscopy (SEM; SNE-ALPHA; SEC, Suwon, Republic of Korea). The samples were spread on carbon tape, and any free unattached dry powder was blown off. The attached dry powders were gold-coated using a G20 sputtering device (GSEM Co., Ltd., Suwon, Republic of Korea) prior to imaging. The particle size distributions (PSD) of the prepared formulations were determined using a laser diffraction particle sizer (Mastersizer 3000e; Malvern Panalytical, Great Malvern, UK) and the wet dispersion method when dispersed in distilled water. The measurement principle is based on the simultaneous multi-angle detection of scattered light. Each measurement was conducted in triplicate, and the mean and standard deviation were calculated.

### 2.5. Powder X-Ray Diffraction

The powder X-ray diffraction (PXRD) patterns of prepared formulations were measured using SmartLab (Rigaku, Tokyo, Japan) with Cu Kα radiation generated at 40 mA and 40 kV. The samples were placed on a silicon plate at 25 °C and 2*θ* scans were collected from 5° to 40°.

### 2.6. Differential Scanning Calorimetry

The thermal properties of different Mg.st hydrate forms were analyzed using differential scanning calorimetry (DSC; Q2000^®^, TA Instruments Ltd., New Castle, DE, USA). The samples were heated from 20 °C to 350 °C at a rate of 20 °C/min under a nitrogen flow of 50 mL/min. For macitentan, DSC analysis was performed over a temperature range of 20 °C to 200 °C, considering its melting point around 130 °C, under the same heating rate and nitrogen flow conditions.

### 2.7. High-Performance Liquid Chromatography Analysis

The simultaneous analysis of MAC and TAD was performed using the Agilent 1200 high-performance liquid chromatography (HPLC) system (Agilent Technologies, Santa Clara, CA, USA) with a 5 μm, C18 column (100 Å, 150 mm × 4.6 mm) from Phenomenex (Torrance, CA, USA). The mobile phase consisted of a 10 mM ammonium acetate aqueous solution, acetonitrile, and methanol at a ratio of 40:12:48 (*v*/*v*/*v*). The flow rate was maintained at 1.0 mL min throughout the analysis. The column temperature was maintained at 35 °C. The detection wavelength was 260 nm, and the injection volume was 10 μL. The mobile phase was used as the sample diluent. The calibration curve for MAC exhibited linearity over the concentration range of 3.15–100 μg/mL (r^2^ = 0.99998), while that for TAD and the calibration was linear in the range of 12.5–400 μg/mL (r^2^ = 0.99997).

### 2.8. Aerodynamic Performance of Prepared MAC and TAD Formulations

The aerosol performance of the prepared formulations was evaluated with the Hamihaler device (Hanmi, Seoul, Republic of Korea) using a next-generation impactor (NGI, COPLEY Scientific, Nottingham, UK), following the USP Chapter <601> specifications for aerosols. A flow rate of 60 L/min was established and confirmed before each experiment using a flowmeter (DFM 2000; COPLEY Scientific, Nottingham, UK). The NGI stage collection plates were coated with silicone oil to prevent particle bouncing and re-entrainment during the test. Three gelatin capsules were manually filled with 30 mg of the prepared formulations.

The mouthpiece was mounted on an induction port and the devices were inserted into it. The experiments were performed at a flow rate of 60 L/min for 4 s, controlled using a flow controller (TPK-R^TM^, COPLEY Scientific, Nottingham, UK). The amount of the drug remaining in the capsule and deposited onto the collection plate in each state was quantified using a validated HPLC method. Aerodynamic cut-off diameters of each stage were determined as 8.06 µm, 4.46 µm, 2.82 µm, 1.66 µm, 0.94 µm, 0.55 µm, and 0.34 µm for stages 1 to 7 at a flow rate of 60 L/min. The emitted dose (ED) was defined as the percentage difference between the initial weight and the remaining weight of the drug in the device and capsule after aerosolization. The fine particle fraction (FPF) was defined as the ability of a particle to reach a respirable region with an aerodynamic size of approximately 5.0 μm or less. The FPF was expressed as the proportion of ED collected during stage 2 through the micro-orifice collector (MOC). This is based on Equations (1)–(3).

(1) $$\mathrm{Emitted}\;\mathrm{dose}\;\left(\mathrm{ED}\right)\%=\frac{\mathrm{Total}\;\mathrm{dose}\;\mathrm{in}\;\mathrm{capsules}\;-\;\mathrm{Drug}\;\mathrm{amount}\;\mathrm{remaining}\;\mathrm{in}\;\mathrm{the}\;\mathrm{capsule}\;\mathrm{and}\;\mathrm{devices}}{\mathrm{Total}\;\mathrm{dose}\;\mathrm{in}\;\mathrm{capsules}}\times 100$$

(2) $$\mathrm{Fine}\;\mathrm{particle}\;\mathrm{fraction}\;\left(\mathrm{FPF}\right)\%=\frac{\mathrm{Total}\;\mathrm{drug}\;\mathrm{amount}\;\mathrm{on}\;\mathrm{stages}\;2\;\mathrm{through}\;\mathrm{MOC}}{\mathrm{Total}\;\mathrm{drug}\;\mathrm{amount}\;\mathrm{on}\;\mathrm{all}\;\mathrm{stages}}\times 100$$

(3) $$\mathrm{Fine}\;\mathrm{particle}\;\mathrm{dose}\;\left(\mathrm{FPD}\right)\mu g=\mathrm{Total}\;\mathrm{drug}\;\mathrm{amount}\;\mathrm{on}\;\mathrm{stages}\;2\;\mathrm{through}\;\mathrm{MOC}$$

Mass median aerodynamic diameter and geometric standard deviation were calculated from the drug mass deposition during the NGI stages. All experiments were performed with *n* = 3.

### 2.9. Statistical Analysis

All statistical analyses were conducted using one-way analysis of variance (ANOVA) followed by Tukey’s post hoc test, as well as *t*-tests, using GraphPad Prism 8 (version 8.4.2; GraphPad Software, San Diego, CA, USA). *p* values < 0.05 were considered statistically significant.

## 3. Results and Discussion

### 3.1. Spray Dried MAC and TAD Formulations

**Figure 1:**
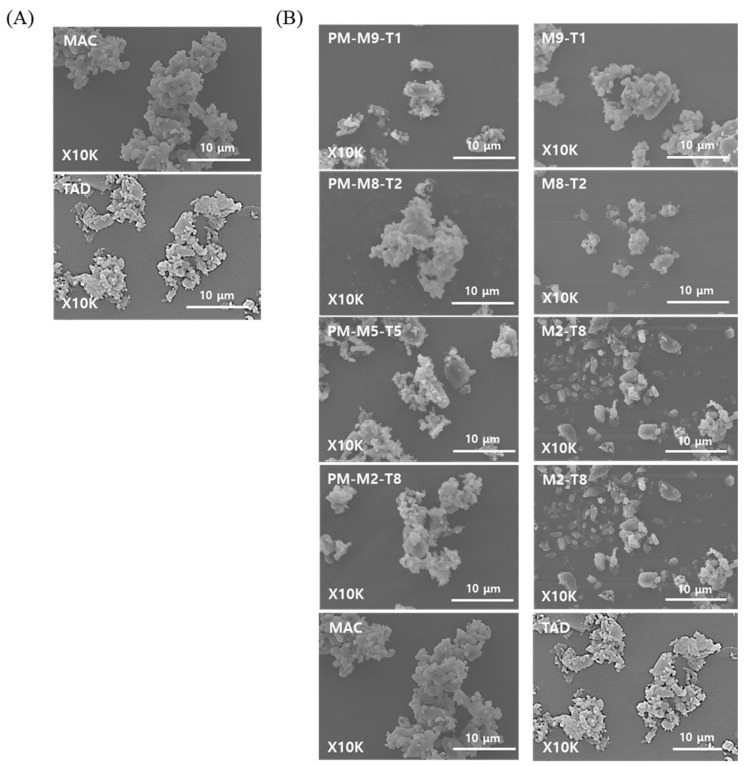
SEM images of the prepared formulations: (**A**) Raw MAC and TAD, and (**B**) Physical mixture (PM) and spray-dried formulation of MAC and TAD.

As shown in Figure 1, the formulation prepared via spray drying the MAC and TAD suspensions exhibited no distinct morphological differences from the physical mixture (PM) of the two components, as observed using SEM. This was due to the low solubility of both drugs, which remained largely undissolved and were sprayed in suspension despite the use of 20% ethanol during atomization. This limited solubility likely maintained the crystallinity of the drugs and minimized particle size reduction during spray drying, resulting in similar particle shapes and sizes between the two formulations. In preliminary solubility tests conducted in 70% ethanol, MAC and TAD have low solubilities of 0.16 ± 0.01 mg/mL and 0.66 ± 0.05 mg/mL, respectively, further confirming that both compounds remain poorly soluble even under ethanol-rich conditions.

The results of the particle size analysis are listed in Table 3. The Dv90 values of the MAC and TAD raw powders were 343.7 ± 30.7 µm and 219.0 ± 6.6 µm, respectively, which differed substantially from the primary particle sizes (<5 µm) observed in the SEM images. This discrepancy was attributed to the strong cohesiveness of the raw powders, which caused the fine particles to aggregate tightly and form large secondary agglomerates. For the physical mixtures, PM-M9-T1, PM-M2-T8, and PM-M1-T9 exhibited relatively small Dv90 values approximately 10 µm, whereas PM-M3-T7 and PM-M5-T5 showed Dv90 values exceeding 100 µm, indicating significant batch-to-batch variability arising from uncontrolled agglomerate formation.

In contrast, the spray-dried formulations exhibited Dv50 values of 2–6 µm and Dv90 values of approximately 10 µm, demonstrating an overall uniform particle size distribution. The spray dried formulations showed a markedly narrower span than that of the physical mixtures, indicating more consistent particle sizes. These findings indicate that preparing the feed as a suspension before spray drying enabled a uniform dispersion of the primary MAC and TAD particles. During atomization, each droplet may have encapsulated a homogeneously distributed mixture of MAC and TAD, and rapid solvent evaporation during drying likely contributed to the formation of secondary particles of uniform sizes [25,26]. The reduced cohesiveness of the spray-dried formulations allowed them to disperse efficiently during particle size measurements, which explains why the resulting PSD values aligned closely with the primary particle sizes observed in the SEM images. These results highlight that suspension-based spray drying, even without complete solubilization of the drugs, can effectively reorganize cohesive primary particles into secondary agglomerates with improved size uniformity and dispersibility.

### 3.2. Aerodynamic Performance of PM and Spray Dried Formulations

**Figure 2:**
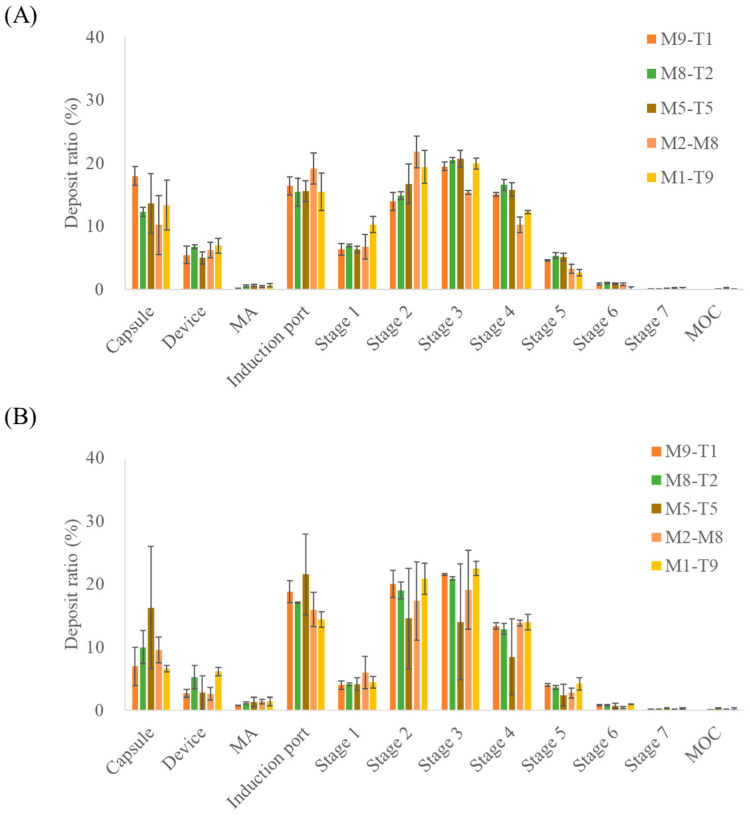
NGI stage deposition profiles of the MAC–TAD combination formulations calculated based on macitentan: (**A**) Physical mixture and (**B**) Spray dried formulations (*n* = 3). MA, mouthpiece adaptor; MOC, micro-orifice collector. The NGI stages correspond to aerodynamic cut-off diameters as follows: Stage 1, 8.06 µm; Stage 2, 4.46 µm; Stage 3, 2.82 µm; Stage 4, 1.66 µm; Stage 5, 0.94 µm; Stage6, 0.55 µm; and Stage 7, 0.34 µm.

The aerosol performance results of the PM and spray dried formulations evaluated based on MAC content and deposition across the NGI stages are presented in Figure 2; the aerodynamic performance data are provided in Table 4. When the physical mixtures were compared with the spray-dried samples, the spray dried formulations exhibited higher mean ED values, indicating that a greater proportion of the total dose was emitted during actuation. In addition, differences were observed in the amount of drug retained within the capsules between the PM and spray dried formulations, as reflected in the ED values. The fine particle fraction of PM and spray dried formulations differed by approximately 5%; however, a substantial difference was observed for the 5:5 ratio, where the FPF values were 72.46 ± 1.07% and 48.91 ± 27.88% for the PM and spray dried formulations, respectively. This discrepancy may be due to enhanced interparticle interactions when the two drug components are present in equal proportions, potentially increasing cohesiveness [27]. Spray dried formulations may exhibit greater agglomeration due to the particle structures or surface characteristics formed during spray drying. This interpretation is supported by the stage deposition results, which showed relatively higher amounts of drug retained in the capsules and deposited in the induction port than in those of the PM formulations. Nevertheless, additional physicochemical characterizations are required to elucidate the underlying mechanisms.

For the PM and spray dried formulations, a decrease in the MAC proportion resulted in a corresponding decrease in FPD, reflecting the reduced amount of drug in the formulation. Similarly to the trend observed for FPF, a large difference between PM and spray drying was noted for the M5-T5 formulation, with FPD values of 1739.05 ± 279.94 μg and 1392.9 ± 784.09 μg, respectively, indicating that the PM formulation exhibited a higher FPD than the spray dried formulation.

**Figure 3:**
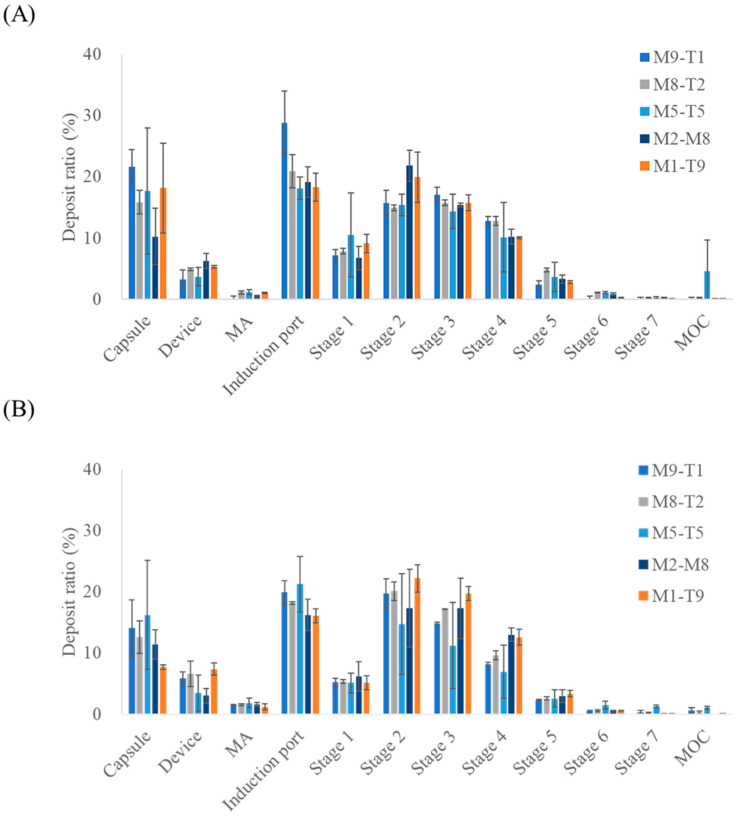
NGI stage deposition profiles of the MAC–TAD combination formulations calculated based on tadalafil: (**A**) Physical mixture and (**B**) Spray dried formulations (*n* = 3). MA, mouthpiece adaptor; MOC, micro-orifice collector. The NGI stages correspond to aerodynamic cut-off diameters as follows: Stage 1, 8.06 µm; Stage 2, 4.46 µm; Stage 3, 2.82 µm; Stage 4, 1.66 µm; Stage 5, 0.94 µm; Stage6, 0.55 µm; and Stage 7, 0.34 µm.

The aerosol performance results of the mixtures and spray dried formulations, assessed based on TAD content, are presented in Figure 3 and Table 5. Similarly to the results obtained for the MAC, the spray dried formulations exhibited higher mean ED values across all composition ratios when evaluated in terms of TAD. In addition, the spray dried formulations consistently demonstrated higher mean ED values than the corresponding PM formulations at all TAD-containing ratios. For PM and spray dried formulations, the FPD increased as the proportion of TAD in the formulation increased, reflecting greater drug content. However, unlike MAC, TAD displayed higher FPD values in spray dried formulations than in its PM counterparts. The M2-T8 sample (3073.45 ± 1312.30 μg) exhibited a higher FPD than the M1-T9 sample (2896.83 ± 531.38 μg), despite containing a lower proportion of TAD. This may indicate that the adhesive interactions between MAC and TAD were stronger than the intrinsic cohesiveness of TAD alone, thereby enhancing its dispersion and aerosolization. In contrast, the adhesion forces between MAC and TAD may have formed at levels comparable to those of the cohesiveness of MAC, leading to a minimal impact on MAC aerosolization behavior [27,28]. Although differences in aerosolization performance suggested altered interparticle interactions, the adhesion and cohesion forces were not quantitatively measured in this study and were inferred indirectly from aerodynamic performance. Future studies using direct measurements (e.g., atomic force microscopy, inverse gas chromatography, and powder rheology) are warranted to validate these assumptions. The differences in the mean FPF across the MAC:TAD ratios were 14.66%, 9.21%, 10.50%, 5.25%, and 4.14% for PMs with 9:1, 8:2, 5:5, 2:8, and 1:9 ratios, respectively. For the spray dried formulations, the corresponding values were 8.48%, 5.46%, 1.54%, 1.69%, and 3.36%, respectively, indicating that spray drying reduced the FPF differences between MAC and TAD compared to the PMs. This suggests that the spray drying process promotes the formation of a greater number of secondary agglomerates relative to PMs, likely due to the co-drying of MAC-TAD droplets during preparation. Thus, the tendency of the two drugs to deposit together during aerosolization is increased. Notably, the selected M2-T8 formulation corresponds to a MAC:TAD ratio of 1:4, which is consistent with the dose ratio used in the approved oral combination (10 mg MAC/40 mg TAD). This compositional alignment supports the rationale for selecting the 2:8 ratio as a representative formulation for further optimization [17]. Furthermore, selecting the spray drying process for a MAC:TAD ratio of 2:8 can enhance the inhalation efficiency of TAD. Thus, the addition of L-leucine was investigated to determine whether the aerosolization performance could be further improved.

### 3.3. Effect of Leucine Ratio on Spray-Dried Formulations

**Figure 4:**
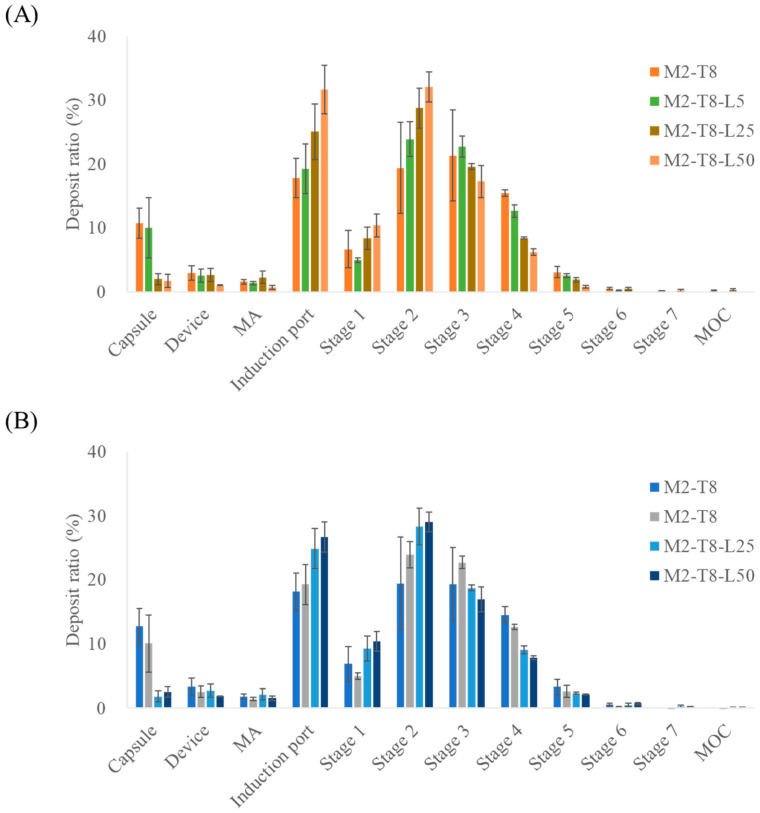
NGI stage deposition of M2-T8 formulations with varying L-leucine ratios: (**A**) macitentan deposition and (**B**) tadalafil deposition (*n* = 3). MA, mouthpiece adaptor; MOC, micro-orifice collector. The NGI stages correspond to aerodynamic cut-off diameters as follows: Stage 1, 8.06 µm; Stage 2, 4.46 µm; Stage 3, 2.82 µm; Stage 4, 1.66 µm; Stage 5, 0.94 µm; Stage6, 0.55 µm; and Stage 7, 0.34 µm.

To evaluate the improvements in inhalation efficiency, L-leucine was incorporated during the preparation of the spray-dried M2-T8 formulation, as shown in Figure 4 and Table 6. Leucine was added at 5, 25, and 50% (*w*/*w*) relative to the total solid content. Based on NGI measurements, MAC and TAD showed statistically significant increases in ED in the M2-T8-L25 and M2-T8-L50 samples. In contrast, the FPF exhibited a slight decrease with the addition of L-leucine.

Among the tested L-leucine concentrations, the M2-T8-L25 formulation exhibited the highest FPD values, representing the amount of drug reaching the deep lung tissue. The FPD values for MAC and TAD were 896.83 ± 45.19 µg and 3587.19 ± 50.04 µg, respectively. The formulation containing 5% L-leucine had FPD values similar to those of the original M2-T8 formulation, suggesting that this concentration was insufficient for L-leucine to exert a meaningful functional effect. In contrast, the increases observed with M2-T8-L25 represented improvements of 31% (MAC) and 17% (TAD) compared with those of M2-T8 without Leu. For M2-T8-L50, an excessive amount of L-leucine appeared to reduce powder dispersibility, leading to increased deposition in the induction port compared with that of M2-T8-L25. These results indicate that a suitable proportion of L-leucine effectively acted as a force control agent, enhancing aerosolization performance [29,30].

The superior performance of the 25% L-leucine formulation can be attributed to the partial surface crystallization of L-leucine during droplet drying. L-leucine is known to accumulate rapidly at the air–liquid interface and form a low-density corrugated shell that reduces the van der Waals attraction between particles. At 25%, this surface shell was sufficiently continuous to enhance dispersibility, but not overly thick to hinder deagglomeration. However, at 50%, an excessively thick L-leucine shell likely increased particle rigidity and interparticle interlocking, reducing deep-lung delivery despite higher emitted doses.

**Figure 5:**
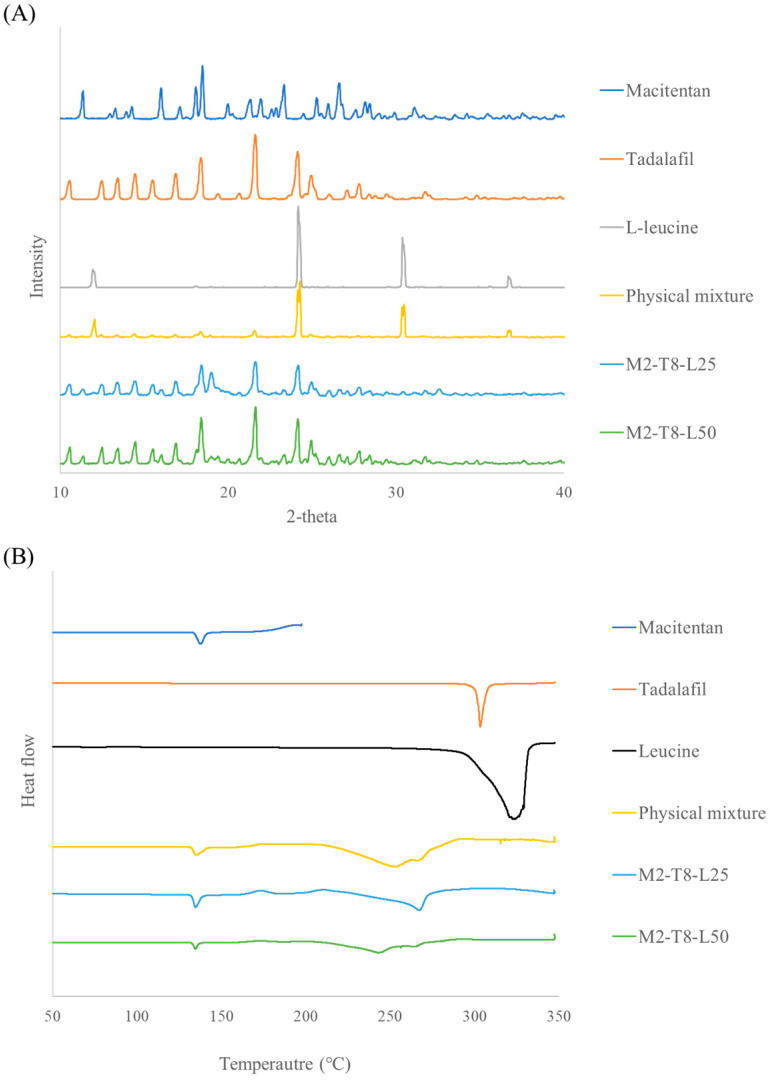
XRD and DSC results of M2–T8–L25 and M2–T8–L50: (**A**) X-ray diffraction patterns and (**B**) Differential scanning calorimetry thermograms.

PXRD analysis was conducted to investigate changes in the crystalline state of the formulations, and the results are shown in Figure 5. In M2-T8-L25 and M2-T8-L50, characteristic diffraction peaks of macitentan (MAC) and tadalafil (TAD) were still observed, indicating that the crystalline structure of the active pharmaceutical ingredients was largely retained after spray drying. However, even in M2-T8-L50, which contained 50% (*w*/*w*) L-leucine, the typical diffraction peaks of L-leucine at approximately 24° and 31° were not detected. This suggests that L-leucine existed in an amorphous or partially crystalline state following the spray-drying process, consistent with previous reports [31,32]. The preferential surface enrichment of L-leucine is considered to modify particle surface properties and reduce interparticle cohesion, thereby contributing to the enhanced inhalation efficiency.

DSC analysis further supported these findings. Endothermic peaks corresponding to the melting of raw macitentan, tadalafil, and L-leucine were observed at approximately 138 °C, 303 °C, and around 320 °C, respectively, with the broad peak of L-leucine attributed to overlapping melting and thermal decomposition. In the physical mixture, the melting peak of macitentan was preserved, whereas the melting event of tadalafil appeared to shift toward a lower temperature (~250 °C). A residual endothermic event near 330 °C was also observed, suggesting the presence of crystalline L-leucine. In contrast, no such thermal events associated with L-leucine were detected in M2-T8-L25 and M2-T8-L50, indicating the transformation of L-leucine into an amorphous or partially crystalline state during spray drying. These DSC results are in good agreement with the PXRD findings.

In this study, the formulation containing 25% L-leucine exhibited the most significant improvement in inhalation performance.

## 4. Conclusions

In this study, the MAC–TAD combination formulations were prepared by physical mixing and spray drying to compare their inhalation performances. Although no notable morphological differences were observed using SEM, particle size analysis revealed that the spray-dried samples exhibited a narrower and more uniform size distribution. Based on in vitro aerodynamic performance evaluation, aerodynamic evaluation further demonstrated improved emitted doses for the spray-dried formulations compared with those of the physical mixtures. Among the MAC:TAD ratios, the M2-T8 formulation showed enhanced aerosolization of TAD after spray drying, despite containing a lower proportion of TAD than other TAD-rich formulations, suggesting that adhesive interactions between MAC and TAD contributed to improved dispersion and aerosolization. The incorporation of L-leucine further improved the inhalation efficiency of M2-T8, with the M2-T8-L25 formulation (25% L-leucine) producing the greatest increase in fine particle delivery for both MAC and TAD. PXRD and DSC analysis indicated that L-leucine remained amorphous or partially crystalline and formed a surface-modifying layer that enhanced dispersibility.

This study provides evidence that a suspension-based co-spray drying process can generate a dual-drug DPI system in which interdrug adhesive interactions selectively enhance the aerosolization of TAD. The asymmetric MAC:TAD ratio of 2:8 demonstrated an unexpected aerodynamic synergy that could not be achieved with either drug alone. Surface engineering with 25% (*w*/*w*) L-leucine further maximized pulmonary deposition, establishing an experimentally validated formulation strategy for an inhaled combination therapy for PAH. These findings introduce a mechanistic and quantitative framework for the rational design of future multidrug DPI systems. However, this study is limited to in vitro aerodynamic characterization, and further in vivo studies are required to confirm lung deposition, pharmacological performance, and safety.

## Figures and Tables

**Table 1:** Component and mixing conditions of MAC and TAD physical mixtures.

Formulation (mg)							
Component	M10-T0	PM-M9-T1	PM-M8-T2	PM-M5-T5	PM-M2-T8	PM-M1-T9	M0-T10
MAC	100	90	80	50	20	10	0
TAD	0	10	20	50	80	90	100
Total	100	100	100	100	100	100	100
Mixing condition							
Rate per minute				3200			
Time (min)				2			

PM, Physical mixture; M, Macitentan; T, Tadalafil.

**Table 2:** Component and spray drying conditions of spay-dried MAC and TAD formulations.

Formulation (mg)								
Component	M9-T1	M8-T2	M5-T5	M2-T8	M1-T9	M2-T8-L5%	M2-T8-L25%	M2-T8-L50%
MAC	450	400	50	100	50	100	100	100
TAD	50	100	50	400	450	400	400	400
Leucine	0	0	0	0	0	26.5	167	500
Total	500	500	500	500	500	526.5	667	1000
Spray drying condition								
Solvent				20% ethanolic solution, 50 mL				
Nozzle size				0.7 mm				
Inlet temperature				120 °C				
Spray air flow				35 m^3^/hour				
Aspirator rate				100%				
Pump rate				10 mL/min				
Nozzle clean interval				2 min				

M, Macitentan; T, Tadalafil; L, Leucine.

**Table 3:** Particle size distribution of the physical mixture and spray-dried formulations of MAC and TAD (*n* = 3).

Formulation		Dv10 (μm)	Dv50 (μm)	Dv90 (μm)	Span
Physical mixture	MAC	7.4 ± 0.2	22.5 ± 0.5	343.7 ± 30.7	15.0 ± 1.6
	TAD	4.5 ± 0.1	15.8 ± 1.0	219 ± 6.6	13.6 ± 0.9
	PM-M9-T1	0.2 ± 0.0	4.2 ± 0.1	11.3 ± 0.3	2.7 ± 0.1
	PM-M8-T2	4.4 ± 0.4	212.0 ± 0.2	349.0 ± 1.4	1.6 ± 0.2
	PM-M5-T5	4.1 ± 0.0	10.7 ± 0.1	181.7 ± 19.6	16.6 ± 1.7
	PM-M2-T8	0.2 ± 0.0	4.2 ± 0.2	11.4 ± 1.4	12.7 ± 0.2
	PM-M1-T9	0.2 ± 0.0	3.8 ± 0.1	10.4 ± 0.9	2.7 ± 1.6
Spray drying	M9-T1	3.0 ± 0.0	6.0 ± 0.1	11.3 ± 0.3	1.4 ± 0.0
	M8-T2	2.7 ± 0.0	4.8 ± 0.1	8.3 ± 0.2	1.2 ± 0.0
	M5-T5	2.9 ± 0.0	5.5 ± 0.0	10.2 ± 0.1	1.3 ± 0.0
	M2-T8	3.0 ± 0.0	5.6 ± 0.0	9.9 ± 0.1	1.2 ± 0.0
	M1-T9	0.2 ± 0.0	2.3 ± 0.1	4.9 ± 0.2	2.0 ± 0.0
	M2-T8-L5	0.2 ± 0.0	3.1 ± 0.1	7.1 ± 0.3	2.2 ± 0.0
	M2-T8-L25	0.2 ± 0.0	4.0 ± 0.2	10.4 ± 0.7	2.5 ± 0.3
	M2-T8-L50	4.4 ± 0.0	11.4 ± 0.7	88.9 ± 15.2	7.4 ± 0.9

PM, Physical mixture; M, Macitentan; T, Tadalafil; L, Leucine.

**Table 4:** Aerodynamic parameters of the MAC–TAD combination formulations calculated based on MAC content (*n* = 3).

Formulation (MAC)	ED (%)	FPF (%)	FPD (μg)	MMAD (μm)	GSD
PM-M9-T1	76.63 ± 2.82	70.22 ± 1.75	3757.09 ± 308.02	3.53 ± 0.11	1.73 ± 0.02
PM-M8-T2	80.97 ± 0.74	71.80 ± 2.44	3141.60 ± 294.78	3.50 ± 0.08	1.76 ± 0.01
PM-M5-T5	81.50 ± 5.52	72.46 ± 1.7	1739.05 ± 279.94	3.60 ± 0.20	1.75 ± 0.01
PM-M2-T8	84.83 ± 5.05	67.13 ± 4.09	778.85 ± 32.05	3.96 ± 0.40	1.77 ± 0.07
PM-M1-T9	79.77 ± 4.75	67.15 ± 3.84	371.96 ± 95.46	4.25 ± 0.21	1.70 ± 0.03
M9-T1	90.36 ± 2.60 **	66.35 ± 1.88	3847.07 ± 136.75	3.78 ± 0.13	1.69 ± 0.03
M8-T2	84.70 ± 0.81 **	67.80 ± 0.58	3432.72 ± 139.09 *	3.79 ± 0.12 *	1.68 ± 0.01 ***
M5-T5	80.95 ± 7.47	48.91 ± 27.88	1392.96 ± 784.09	4.23 ± 0.68	1.78 ± 0.10 *
M2-T8	87.82 ± 1.13	61.25 ± 15.60	685.05 ± 301.83	3.81 ± 0.08	1.75 ± 0.14
M1-T9	87.31 ± 0.56	72.24 ± 2.15	316.64 ± 60.41	3.77 ± 0.20 *	1.70 ± 0.03

Significance levels were as follows: *, *p* < 0.05; **, *p* < 0.005; ***, *p* < 0.001 (*t*-test). All comparisons were performed relative to the PM-each ratio. PM, Physical mixture; M, Macitentan; T, Tadalafil.

**Table 5:** Aerodynamic parameters of the MAC–TAD combination formulations calculated based on tadalafil (*n* = 3).

Formulation (TAD)	ED (%)	FPF (%)	FPD (μg)	MMAD (μm)	GSD
PM-M9-T1	75.14 ± 4.19	55.56 ± 5.44	231.79 ± 50.72	4.28 ± 0.21	1.59 ± 0.05
PM-M8-T2	79.35 ± 2.02	62.59 ± 2.95	635.33 ± 89.99	3.75 ± 0.08	1.92 ± 0.01
PM-M5-T5	78.70 ± 11.68	61.96 ± 11.19	1141.07 ± 288.70	4.18 ± 1.40	2.12 ± 0.24
PM-M2-T8	83.65 ± 5.44	61.88 ± 4.01	2396.34 ± 242.29	4.35 ± 0.32	1.84 ± 0.08
PM-M1-T9	76.63 ± 7.16	63.01 ± 2.28	2658.39 ± 371.14	4.47 ± 0.26	1.81 ± 0.04
M9-T1	80.05 ± 3.87	57.87 ± 1.89	498.41 ± 29.14 **	4.35 ± 0.18	1.74 ± 0.07 *
M8-T2	80.85 ± 1.03	62.34 ± 0.91	826.65 ± 49.72	4.19 ± 0.15	1.74 ± 0.02
M5-T5	80.38 ± 6.52	47.37 ± 23.41	1467.59 ± 678.47 *	3.96 ± 0.31 *	2.16 ± 0.04 ***
M2-T8	85.67 ± 1.30	59.56 ± 15.82	3073.45 ± 1312.30	3.91 ± 0.12	1.79 ± 0.09
M1-T9	84.96 ± 0.92	68.88 ± 2.03 *	2896.83 ± 531.38	4.04 ± 0.26	1.75 ± 0.03

PM, Physical mixture; M, Macitentan; T, Tadalafil. Significance levels are indicated as follows: * *p* < 0.05, ** *p* < 0.005, and *** *p* < 0.001 (*t*-test). All comparisons were performed relative to the PM-each ratio.

**Table 6:** Aerodynamic parameters of the M2-T8 with different leucine ratio (*n* = 3).

Formulations		ED (%)	FPF (%)	FPD (μg)	MMAD (μm)	GSD
M2-T8(-L0)	MAC	87.82 ± 1.13	61.25 ± 15.60	685.05 ± 301.8	3.81 ± 0.08	1.75 ± 0.14
	TAD	85.67 ± 1.30	59.56 ± 15.82	3073.45 ± 1312.3	3.91 ± 0.12	1.79 ± 0.09
M2-T8-L5	MAC	87.87 ± 5.70	67.71 ± 4.73	720.62 ± 92.4	4.1± 0.08	1.65 ± 0.03
	TAD	88.3 ± 5.26	63.91 ± 3.81	3086.31 ± 421.21	4.42 ± 0.02 **	1.75 ± 0.04
M2-T8-L25	MAC	95.52 ± 1.46 !	59.88 ± 3.30	896.83 ± 45.19	4.71 ± 0.16 !!	1.63 ± 0.02
	TAD	95.64 ± 1.47 **	59.53 ± 1.47	3587.19 ± 50.04	4.72 ± 0.21 ***	1.67 ± 0.02
M2-T8-L50	MAC	97.38 ± 1.00 !	63.47 ± 4.17	553.79 ± 60.4	5.29 ± 0.26 !!!	1.53 ± 0.02 !!
	TAD	95.81 ± 0.83 **	56.62 ± 3.69	2453.88 ± 205.36	4.95 ± 0.18 ***	1.69 ± 0.05

! *p* < 0.05, !!/** *p* < 0.005, !!!/*** *p* < 0.001; One-way ANOVA. Comparison with (!) the MAC of M2-T8-L0 and (*) with the TAD of M2-T8-L0.

## Data Availability

The data presented in this study are included in the article. Further inquiries can be directed to the corresponding author.

## Abbreviations

The following abbreviations are used in this manuscript:

DPI	Dry powder inhalation
ED	Emitted dose
ERA	Endothelin receptor antagonist
PSD	Particle size distribution
FPD	Fine particle dose
FPF	Fine particle fraction
HPLC	High-performance liquid chromatograph
MAC	Macitentan
MMAD	Mass median aerodynamic diameter
MOC	Micro-orifice collector
NGI	Next generation impactor
PAH	Pulmonary arterial hypertension
PDE-5	Phosphodiesterase-5
PM	Physical mixture
PXRD	Powder X-ray diffraction
TAD	Tadalafil
